# Feasibility and Outcomes of Early Oral Feeding After Total Gastrectomy for Cancer

**DOI:** 10.1007/s11605-014-2720-0

**Published:** 2014-12-18

**Authors:** Marek Sierzega, Ryszard Choruz, Szymon Pietruszka, Piotr Kulig, Piotr Kolodziejczyk, Jan Kulig

**Affiliations:** First Department of Surgery, Jagiellonian University Medical College, 40 Kopernika Street, 31-501 Krakow, Poland

**Keywords:** Gastrectomy, Gastric cancer, Oral feeding, Complications, Enhanced recovery after surgery

## Abstract

**Background:**

Little data are available supporting the feasibility and safety of early oral feeding in patients after total gastrectomy. The aim of this study was to analyze the potential applicability of early provision of oral diet in these settings.

**Methods:**

Medical records of 353 patients who underwent total gastrectomy for gastric cancer between 2006 and 2012 were retrospectively analyzed. Early oral feeding was defined as clear liquid diet on postoperative day (POD) 1 followed by gradual introduction of solid diet on POD 2 to 3. Late oral feeding was defined as initiation of liquid diet from POD 4 to 6 and gradually advancing to solid diets.

**Results:**

Early oral feeding was implemented in 185 of 353 (52 %) patients. Prompt provision of food did not increase the risk of anastomotic failure (odds ratio 0.924, 95 % confidence interval 0.609–1.402, *P* = 0.709). The number of reoperations and in-hospital mortality rates was unaffected by the timing of nutritional intervention. Early feeding tended to be associated with fewer surgical (15 vs 24 %, *P* = 0.027) and general (8 vs 23 %, P < 0.001) complications. However, subsequent multivariate regression models failed to confirm significant correlations between timing of oral meals and postoperative morbidity.

**Conclusion:**

Our findings suggested that early oral feeding is feasible and safe after total gastrectomy for gastric cancer. However, benefits of such early nutritional interventions require further studies.

## Introduction

For many decades, a “nil-by-mouth” policy has been commonly applied as a surgical dogma after gastrointestinal procedures.^1^,^2^ The long-held belief that nutritional interventions should be withheld until bowel function has resumed was subsequently challenged by several randomized clinical trials (RCTs) on enteral nutrition. Meta-analyses evaluating these studies have definitively confirmed that early provision of enteral diet via nasoenteral or jejunostomy tubes is feasible, safe, and may be beneficial for the patients.^3^–^5^

Observations from RCTs on enteral nutrition persuaded many surgeons to allow normal food by mouth after major gynecologic and colorectal operations.^4^,^6^ However, despite experimental evidence suggesting that oral feeding accelerates upper gastrointestinal anastomotic healing, most surgeons are still reluctant to apply such an early intervention following resections of gastric cancer for fear of compromising the integrity of an anastomosis.^7^ This is particularly evident for total gastrectomy, as an esophagojejunostomy is considered more likely to leak than a gastrojejunostomy. These ambiguities were not solved by studies adopting enhanced recovery after surgery (ERAS) protocols as they presented data for inhomogeneous populations of patients subject to different operations and the number of total gastrectomies varied from 5 to 50.^8^–^11^ A recent questionnaire survey in major digestive surgical centers in Scotland, the Netherlands, Denmark, Sweden, and Norway demonstrated that a nil-by-mouth policy is relatively common after gastric resections even for 3–4 days, and eating at will is generally allowed by the first postoperative day (POD) only in 20 % of the responding centers.^12^ A similar survey carried out in 2009 recruiting nearly 4000 gastric cancer patients in 66 centers from 18 countries revealed that oral fluids following total gastrectomy were started on median POD 4 (range 1 to 6) and solids on POD 6 (range 4 to 8).^13^

A structured recovery pathway for all patients subject to gastrointestinal surgery was initiated in 2006 at our academic tertiary surgical center. Early oral feeding was subsequently incorporated in the protocol and constituted an important element of postoperative care. Given the paucity of published data related to such interventions after total gastrectomy for cancer, the aim of this study was to analyze the feasibility, safety, and potential benefits of early provision of oral diet in this patient population.

## Materials and Methods

An electronic database of all patients with gastric cancer treated between 1977 and 2012 at our academic tertiary surgical center was reviewed. All relevant data, including demographics, clinical findings, details of surgical procedures, and histopathological parameters, was collected prospectively using standardized forms of the Polish Gastric Cancer Study Group and stored in a dedicated electronic database. The extent of surgery, definitions for lymph node dissection, and tumor staging were adapted to the recent guidelines.^14^,^15^ During the postoperative period, all patients were observed for both surgical and non-surgical complications. Anastomotic leakage was diagnosed radiologically (extravasation of the oral contrast medium at the anastomotic site or with fistulography) or clinically (discharge of saliva or gastrointestinal content through a drain or at relaparotomy). Abdominal abscess was defined as collection of fluid diagnosed with US/CT and positive cultures obtained by percutaneous drainage or at reoperation. Non-abscess abdominal fluid collection was defined as a collection of fluid measuring ≥3 cm in diameter demonstrated by transabdominal US or CT scan. Other complications were defined as previously reported.^16^ Postoperative mortality was defined as any death during the hospital stay after surgery.

**Fig. 1:**
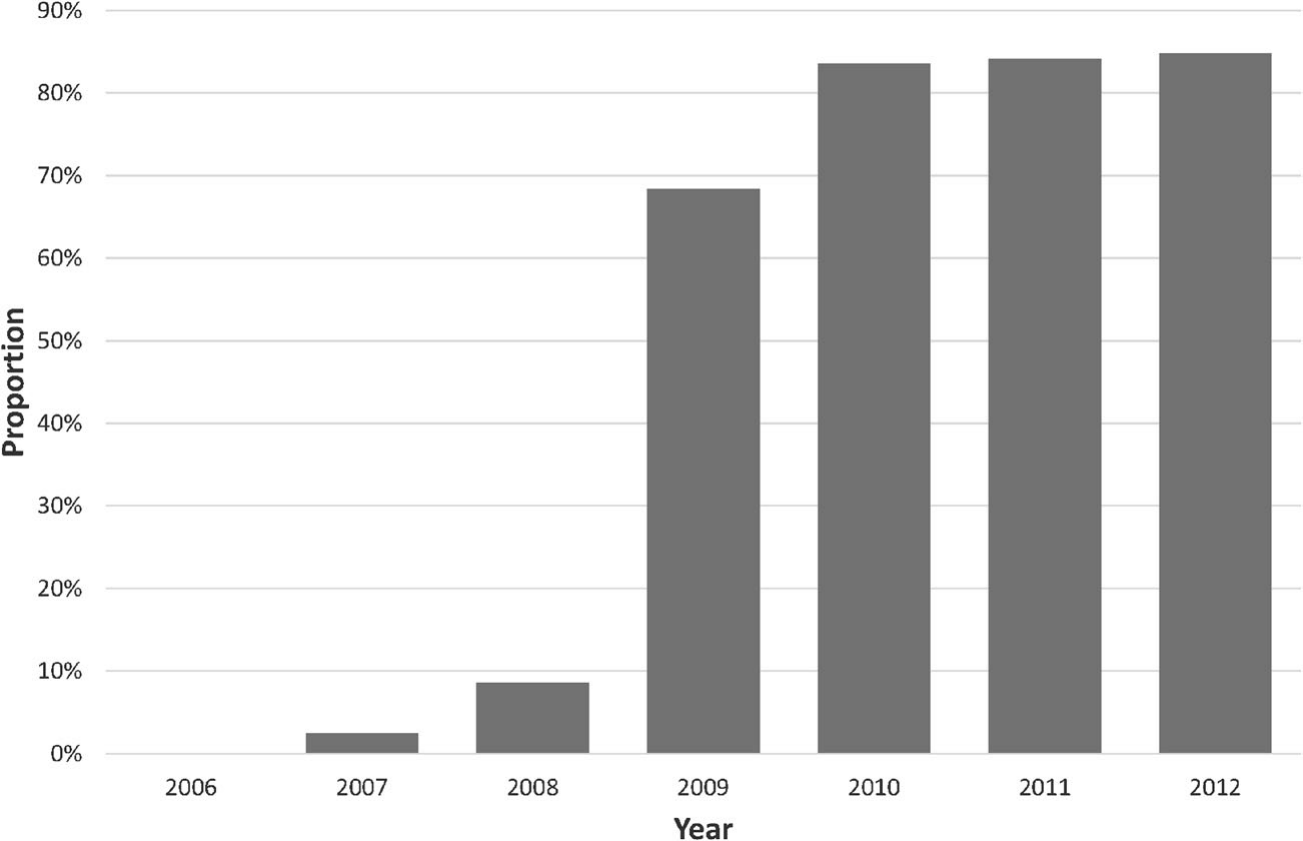
The annual proportion of patients receiving early oral diets

The present study was limited to a period from 2006 to 2012 when a uniform clinical pathway was implemented for all patients subject to gastric resections (Table 1). All procedures were carried out by or under supervision of five senior consultant surgeons experienced in upper gastrointestinal surgery with a total annual caseload of about 100 patients with gastric resections for cancer. During the operation, reconstruction was performed with a Roux-en-Y esophagojejunostomy through a transabdominal or thoraco-abdominal approach depending on the extent of the necessary esophageal resection. All esophagojejunal anastomoses were performed with circular stapling devices. Closed non-vacuum abdominal drains were routinely placed. No routine tests (clinical or radiological) were carried out intra- or postoperatively to determine the integrity of the anastomosis. Initially, patients received oral fluids starting on POD 4, followed by a soft diet (thin purée six times a day) on day 5 and regular solid diet thereafter. From 2009, our unit protocol was modified (Fig. 1) by introducing liquids already on POD 1, followed by a soft diet on POD 2, and solid foods on day 3. There was no predefined target volume of oral diets to be reached. Other elements of the postoperative pathway remained unaltered throughout the study period, including discharge criteria, i.e., no postoperative complications, ability to ambulate without assistance, tolerable pain with oral analgesics, ability to take more than 75 % of a given meal, and a willingness to go home. For the purpose of this study, patients were retrospectively divided into an early oral feeding group (soft diet on POD 2 or 3) and late oral feeding group (initiation of liquid diet from POD 4 to 6). Postoperative outcomes in both groups were compared by an intent-to-treat principle, i.e., patients who received at least one oral meal on POD 1–3 were assigned to the early feeding group and on POD 4–6 to the late feeding group. Patients who died without any oral feeding on POD 1 to 6 and those starting their diet on POD 7 or later due to any reasons were excluded.

**Table 1:** Components of perioperative care

Timing	Item
Preoperative	General medical optimisation and counselling Nutritional support in significantly malnourished patients Normal diet until midnight No oral bowel preparation Preanesthetic medication without long-acting sedatives Anti-thrombotic prophylaxis (LMWH and mechanical measures) Antimicrobial prophylaxis 30–60 min before skin incision
Intraoperative	Combination of mid-thoracic epidural analgesia and general anesthesia Prophylaxis of hypothermia by using cutaneous warming Avoidance of salt and water overload Midline incision of a length sufficient to ensure good exposure One or two abdominal drains routinely placed Naso-jejunal feeding tube in malnourished patients No nasogastric intubation Patients transferred to anesthesia recovery room
Postoperative	Epidural analgesia combined with intravenous analgesia Pharmacological prophylaxis of postoperative nausea and vomiting Transurethral catheters removed on POD 1 or 2 unless otherwise indicated Naso-jejunal tube feeding in malnourished patients Anti-thrombotic prophylaxis (LMWH) continued until discharge Drain removal on POD 4 to 5 unless otherwise indicated Early mobilization starting on POD 1

### Statistical Analysis

The differences in proportions between groups were evaluated using the chi-square test, and the Mann–Whitney *U* test was used to detect differences in quantitative variables. Potential risk factors for postoperative complications were evaluated by univariate analysis using cross-tabulations and a stepwise logistic regression model with a probability to enter the model of 0.1 and a probability to leave of 0.05. The following factors were analyzed: sex, age (continuous variable), year of surgery (2006–2009 vs 2010–2012), American Society of Anesthesiologists (ASA) physical status (0–1 vs 2–3), preoperative body weight loss (<10 vs ≥10 %), body mass index (BMI <25 vs ≥25), preoperative albumin level (<3.5 vs ≥3.5 g/dL) and lymphocyte count (<1500 vs ≥1500 per mm^3^), pre-existing diseases (cardiovascular, respiratory, renal, diabetes mellitus, and cirrhosis), extent of lymphadenectomy (D1, D2, and D2+), splenectomy, pancreatectomy, tumor stage (I–II vs III–IV), curability of resection (R0 vs R1-2), operative time (continuous variable), and need for autologous blood transfusion. Additionally, the presence of pancreatic fistula and anastomotic leakage was evaluated as potential predictors for systemic complications. Statistical analysis was performed using the IBM® SPSS® Statistics v.21 (IBM Corp., Armonk, NY) software package, and two-sided *P* values of less than 0.05 were considered to indicate statistical significance.

## Results

### Study Population

Between 2006 and 2012, a total of 725 patients underwent gastric resections for cancer, including 396 subject to total gastrectomy. Forty-three patients were excluded from the current analysis due to death without any oral feeding (*n* = 12), and oral diet started on POD 7 or later (immediate postoperative ICU transfer due to respiratory or circulatory failure, *n* = 31). Five of these 43 patients had anastomotic leaks. Therefore, the final study population consisted of 353 patients who received at least a single oral meal on POD 1 through 6, including 251 males and 102 females with a median age of 63 years (range 26 to 84). With the exception of a higher prevalence of chronic pulmonary disorders in the late feeding group, both populations were well balanced in terms of preoperative clinical and laboratory parameters (Table 2). The extent of surgical intervention was also similar; however, splenectomy was less common in the early feeding group.

**Table 2:** Baseline characteristics of study population

	Early feeding (*n* = 185)	Late feeding (*n* = 168)	*P*
Age, years (median, IQR)	63 (55–72)	64 (53–73)	0.790^a^
Female/male, *n*	54/131	48/120	0.898^b^
Comorbidities, *n* (%)			
Cardiovascular	72 (39)	72 (43)	0.452^b^
Respiratory	7 (4)	20 (12)	0.005^b^
Diabetes mellitus	28 (15)	22 (13)	0.583^b^
Renal	5 (3)	4 (2)	0.848^b^
Cirrhosis	3 (2)	0 (0)	0.097^b^
ASA physical status, 1–2/3–4 (*n*)	152/33	128/40	0.166^b^
Body mass index (median, IQR)	25.3 (22.2–28.9)	24.7 (21.7–27.8)	0.259^a^
Preoperative body weight loss, % (median, IQR)	12.5 (6.9–19.1)	12.9 (8.1–18.9)	0.498^a^
Serum albumin, g/dL (median, IQR)	41 (37–45)	40 (37–43)	0.073^a^
Lymphocytes, per mm^3^ (median, IQR)	1520 (1100–2100)	1400 (1200–2300)	0.130^a^
Preoperative chemotherapy, *n* (%)	46 (25)	41 (24)	0.920^b^
Tumor size, mm (median, IQR)	138 (112–153)	108 (80–149)	0.045^a^
Tumor stage (AJCC 2010), *n* (%)			
I	24 (13)	13 (8)	0.167^b^
II	31 (17)	20 (12)	
III	95 (51)	92 (54)	
IV	35 (19)	43 (26)	
Curative resection (R0), *n* (%)	98 (53)	97 (58)	0.368^b^
Lymph node dissection (JGCA), *n* (%)			
D1	15 (8)	12 (7)	0.892^b^
D2	113 (61)	108 (64)	
D2+	57 (31)	48 (29)	
Thoracoabdominal resection, *n* (%)	11 (22)	9 (38)	0.811^b^
Splenectomy, *n* (%)	40 (22)	64 (38)	<0.001^b^
Pancreaticosplenectomy, *n* (%)	7 (4)	13 (8)	0.108^b^
Operative time, min (median, IQR)	180 (150–210)	195 (165–235)	0.110^a^
Need for blood transfusion, *n* (%)	54 (29)	44 (26)	0.529^b^
Postoperative enteral feeding, *n* (%)	148 (80)	153 (91)	0.003^b^

*AJCC* American Joint Committee on Cancer Classification 2010, *JGCA* Japanese Gastric Cancer Association Classification 2010, *IQR* interquartile range

^a^Mann–Whitney *U* test

^b^Chi-square test

### Postoperative Complications

The overall morbidity rate was 27 % (97 of 353 patients) and was significantly higher in the late feeding group (36 vs 20 %, *P* < 0.001). However, the number of reoperations and in-hospital mortality rates was unaffected by the timing of nutritional intervention (Table 3). Early oral feeding was associated with significantly fewer surgical complications (15 vs 24 %, *P* = 0.027), and this was attributed to the less common incidence of abdominal fluid collections. However, of particular importance was the fact that early provision of food did not increase the risk of anastomotic failure (odds ratio [OR] 0.924, 95 % confidence interval [CI] 0.609–1.402, *P* = 0.709). The median interval from surgery to diagnosis of anastomotic leak was 5 days without any differences in presentation between early and late feeding groups. General complications were significantly higher in patients receiving late feeding (8 vs 23 %, *P* < 0.001) due to the more common incidence of respiratory morbidity. Causes of postoperative mortality were comparable between both groups and related to anastomotic leakage (*n* = 6), cardiocirculatory failure (*n* = 4), and necrosis of the transverse colon (*n* = 1).

**Table 3:** Postoperative complications

	Early feeding (*n* = 185)	Late feeding (*n* = 168)	*P* ^a^
Surgical complications	27 (15 %)	40 (24 %)	0.027
Wound infection	12	12	0.806
Abdominal fluid collection	8	20	0.008
Anastomotic leakage	7	2	0.122
Pancreatic fistula	6	11	0.147
Abscess	3	8	0.089
Peritonitis	3	1	0.362
Ileus	3	1	0.362
Abdominal bleeding	1	1	0.945
Duodenal stump leakage	1	4	0.143
Other	3	4	0.609
General complications	15 (8 %)	38 (23 %)	<0.001
Pneumonia	9	24	0.003
Respiratory failure	6	17	0.009
Heart failure	6	11	0.147
Renal failure	2	4	0.345
Urinary tract infection	1	4	0.143
Sepsis	1	5	0.077
Liver failure	0	2	0.136
Other	3	4	0.609
Reoperations	11	11	0.815
Mortality	5	6	0.639

^a^Chi-square test; some patients had two or more complications

ASA class 3–4, BMI >25, splenectomy, and late oral feeding were found to be potential predictors of respiratory complications by univariate analysis. Subsequent regression analysis identified only two independent risk factors, i.e., higher ASA class (OR 9.35, 95 % CI 4.71–18.55, *P* = 0.001) and splenectomy (OR 2.40, 95 % CI 1.19–4.82, *P* = 0.014). Splenectomy, need for blood transfusion, delayed oral feeding, pancreatic fistula, and anastomotic leakage were associated with a higher incidence of abdominal fluid collections. Among those, only pancreatic fistula (OR 10.77, 95 % CI 3.88–29.92, *P* < 0.001), anastomotic leakage (OR 18.25, 95 % CI 6.38–52.20, *P* < 0.001), and need for blood transfusion (OR 2.12, 95 % CI 1.01–4.91, *P* = 0.048) were related to collections in the regression model.

### Functional Parameters and Hospital Stay

Patients receiving early oral feeding had shorter time to the first flatus (median 2 days (interquartile range [IQR] 1–3) vs 3 (IQR 2–5), *P* = 0.020) and defecation (median 3 days (IQR 2–4) vs 5 days (IQR 3–7), *P* = 0.001). However, the proportion of patients who required temporary withdrawal of oral diet due to poor tolerance was similar in the early (13 of 185) and late (9 of 168) feeding protocol (*P* = 0.517). Twenty-five patients prematurely discontinued nasojejunal feeding due to tube displacement or poor tolerance of the administered diet. However, no patient had replacement of nasojejunal tube. Total parenteral nutrition was necessary in 42 patients, evenly distributed between both study groups, due to postoperative complications (*n* = 26), marked preoperative malnutrition (*n* = 10), and poor tolerance of oral/enteral nutrition (*n* = 6). The median postoperative hospital stay in patients receiving early oral feeding (7 days, IQR 6–8) was significantly shorter than for late feeding (8 days, IQR 7–15, *P* < 0.001). Differences in the median hospitalization time favoring early feeding were preserved even after exclusion of the two most common groups of complications, i.e., respiratory morbidity (median 7 days (IQR 6–7) vs 8 days (IQR 7–10), *P* < 0.001) and abdominal fluid collections (median 7 days (IQR 6–8) vs 8 days (IQR 7–11), *P* < 0.001). The median follow-up time after discharge was 46 months (IQR 32–54) without significant differences between early (median 46 months) and late (median 45 months) feeding. Anastomotic strictures were found in 17 (5 %) of patients and were not related to timing of oral feeding.

## Discussion

Early provision of oral diet did not increase postoperative morbidity, including compromised integrity of an esophagojejunal anastomosis, in this homogenous population of Western patients undergoing total gastrectomy for cancer. Therefore, such nutritional intervention can be safely adapted to accelerated patient recovery protocols.

For many decades, the fear of anastomotic failure has deterred surgeons from allowing early oral intake of food after gastrointestinal surgery. Recent systematic reviews suggested that accelerated oral feeding is feasible and safe; however, previous studies focusing on upper gastrointestinal surgery recruited heterogeneous populations of patients subject to various esophageal, gastric, hepatic, or pancreatic operations.^17^ Three RCTs evaluated early oral feeding as a component of ERAS specifically among patients subject to gastric resections due to cancer.^9^,^10^,^18^ All three trials demonstrated shortened hospital stay without impaired postoperative morbidity and mortality. However, despite the encouraging results of these studies, they poorly reflect the clinical scenario of total gastrectomy in a Western population for several reasons. Firstly, only Wang et al. and Liu et al. recruited small groups of patients subject to total gastrectomy, and their numbers in the ERAS protocols were 7 and 13, respectively.^9^,^10^ Secondly, the trial of Chen Hu et al. incorporated patients undergoing laparoscopic and open distal gastrectomy, which further complicated interpretation of data. Thirdly, all three studies were carried out in Asian countries excluding patients with significant comorbidities. All these ambiguities raise doubts related to the use of early oral feeding after total gastrectomy that is further supported by the current consensus guidelines of the ERAS® Society.^19^ Although the society suggests early oral meals at will from POD 1, the authors emphasize that data from Western populations are scant and the recommendation grade is weak.

A uniform clinical pathway, compliant with most recommendations for an ERAS protocol in gastric surgery, was adopted postoperatively for all the patients in this study.^19^ The only variable changing over time was the onset of oral feeding; thus, we were able to specifically evaluate the effects of early oral nutrition. Using a well-defined population of patients undergoing total gastrectomy for cancer, we have specifically demonstrated that early oral provision of food did not increase morbidity rates, including the risk of anastomotic failure. Although results of the univariate analysis suggested that the risk of some complications could be reduced by early oral provision of food, this was not confirmed by multivariate models. Therefore, such findings should be considered a coincidence rather than a causal relationship.

Quite recently, two prospective observational studies have demonstrated that liquid diet was well tolerated starting on POD 2 after distal gastrectomy.^20^,^21^ The feasibility of early postoperative oral feeding was also confirmed in an RCT by Lassen et al. allowing normal food at will from the first day after surgery, including 39 patients subject to total gastrectomy.^22^ Although this suggested that early oral feeding may be tolerated by patients following gastric resections, appropriate provision of calories and nutrients in this way was not well documented to date. It is well recognized that actual postoperative intake of oral diets is less than 100 % in most cases increasing the malnutrition-related risk in already nutritionally depleted patients with upper gastrointestinal malignancies.^2^,^20^ Most participants of this study were at increased nutritional risk; therefore, enteral tube feeding was used in about 85 % of cases according to the European Society for Clinical Nutrition and Metabolism (ESPEN) recommendations for malnourished surgical patients.^23^ This ensured that the potential poor tolerance of oral diet would not preclude the necessary provision of calories and nutrients. Although about 6 % of patients in this study required temporary withdrawal of oral diet due to poor tolerance, daily monitoring of oral intake showed that the goal of 75 % was achieved after 3 to 4 days of oral feeding. This suggests that early oral feeding should be considered as a sufficient nutritional intervention in well-nourished patients. However, it is not known whether such diets are adequate as the sole postoperative provision of nutrients in nutritionally depleted subjects.

Due to the drawbacks inherent in cohort studies, this report has some limitations. The analysis was not planned as a formal ERAS protocol, and thus, not all elements of the perioperative pathway could be standardized, e.g., length of skin incision or early ambulation distance. Despite the fact that all the data were collected prospectively using standardized forms, the lack of randomization and retrospective analysis is potentially associated with selection bias among patients treated in a high volume center as suggested by low rates of anastomotic failures. To minimize this effect, all patients who had oral diets postponed to POD 7 or later due to immediate postoperative complications precluding the possibility of receiving early oral feeding were excluded from the analysis. Another potential source of selection bias is the hypothesis that only those patients who feel well were willing to eat early. However, at least two important facts argue against such a simple assumption. Firstly, since 2009, all the patients were actively encouraged to start early oral intake of food and this was achieved in about 85 % of cases during the recent years (Fig. 1). Secondly, as most complications occurred much later than the anticipated date of early feeding (POD 2 to 3), they could not be responsible for postponing nutritional intervention.

## Conclusion

In conclusion, the present study provides significant evidence supporting the safety and feasibility of early oral feeding in highly selected patients undergoing total gastrectomy for cancer. However, a randomized clinical trial initiated recently (NCT01962519) is still desirable to objectively define whether this may have practical implications in terms of shortened hospital stay and reduced postoperative morbidity.
